# How do career promotion policies affect research publications and open access?

**DOI:** 10.12688/openreseurope.14921.1

**Published:** 2022-08-22

**Authors:** Nancy Pontika, Bikash Gyawali, Antonia Corriea, Helene Brinken, David Pride, Matteo Cancellieri, Petr Knoth

**Affiliations:** 1 Knowledge Media Institute, The Open University, Milton Keynes, MK7 6AA, UK; 2 Big Data Analytics Group, University of Hull, Hull, HU6 7RX, UK; 3 Open Science, Repositories and Scientific Information Office, University of Minho, Minho, Portugal; 4 Göttingen State and University Library, University of Göttingen, Göttingen, Germany

**Keywords:** Open Science, promotion, review, tenure, policies, career development, data set, academic progression.

## Abstract

We present a novel dataset which enables quantitative analysis of the relationship between institutional support for Open Science and research performance. We analysed promotion, review, and tenure policies (PRT) from institutions originating from seven countries and combined them with bibliographical data from the outputs generated by each institution. The data were normalised and evaluated against Open Science and Responsible Research and Innovation (RRI) indicators, to enable comparisons and easy machine readable access. The significance of this dataset lies in its potential to answer a range of questions that are key to the understanding of what motivates academics with regards to their research practices and publishing behaviors, using various indicators, including the MoRRI ^1^. To our knowledge, this collection constitutes one of the first efforts in delivering a large machine readable dataset enabling quantitative analysis on these aspects, as much work in this area has been carried out only through surveys and qualitative analysis.

## 1 Introduction

We present a new promotion, review, and tenure policy (PRT) dataset which enables quantitative analysis of the relationship between institutional support for Open Science ^1^, represented by promotion, review, and tenure criteria in policy documents, and research behaviour and performance. The dataset is composed of: 1) a collection of PRT institutional policies (143 in total) from seven countries (Austria, Brazil, Germany, India, Portugal, the United Kingdom [UK], and United States[USA]); and 2) a corpus of scholarly research outputs processed from Microsoft Academic Graph (MAG) ^2^, a freely available database of scholarly information about research and CORE ^2^, the world’s largest aggregator of Open Access ^3^ content.

The dataset was used in the Observing and Negating Matthew Effects in Responsible Research and Innovation Transition(ON-MERRIT ^4^), an EU-funded project. The paper describes the dataset we collected and it highlights the difficulties in compiling the data required to judge the efficacy of institutional promotional policies. This work leads to the potential benefits of gathering policy information in a standardised, machine-readable way.

## 2 Dataset significance

This paper presents a unique dataset that enables quantitative analysis of mapped links between criteria for academic progression, as defined in promotion policies of academic institutions, and the productivity of academics, as measured by their research outputs and Open Science practices. Such data was previously available in an unstructured way and in a distributed fashion. Here we are curating and processing it into a structured machine-readable dataset in a centralised collection to enable quantitative analysis. Multiple factors can influence academic progression and tenure, such as institutional strategies and culture; however we focus on Open Science and Responsible Research and Innovation (RRI) ^5^ (RRI) factors and seek data to support this study. We document universities’ adoption of Open Science and RRI by assessing various indicators relating to their research performance and assessment. This includes number of citations, journal metrics, peer review, publication quality, etc. In addition, some MoRRI indicators ^1^ are also examined, for example, gender of reviewers, citizen science, public engagement, etc. More specifically, this dataset consists of two subsets linked via an institution entity:

1. **Promotion, review, and tenure (PRT) dataset:** a CSV file with true/false values for the presence/absence of the indicators in the PRT policies. Each institution has a matching id extracted from the research papers dataset (see below).
2. **Research papers dataset:** a large corpus of universities’ scholarly publication records extracted from the MAG database and processed for linking it to the corresponding universities’ promotion policies. It collectively encodes the data on research papers as well as the career profile of their authors as determined from MAG. Other enrichment from external data sources, such as the information on open access status of publications as given by the CORE Discovery service, are also included.

The PRT and the research papers subsets can be used to explore the current situation across the seven aforementioned countries. The collected PRT documents are originally in three languages: English, German, and Portuguese. This is due to the native language spoken at these institutions. The analysis of the promotion policies was therefore undertaken by native speakers. The research papers dataset is fully in English.

Further to the contributions mentioned above, this paper calls for machine accessibility of PRT policies demonstrating the utility of linking them to other scholarly datasets, enabling quantitative analysis of policy instruments and their likely effects. Such data are needed to improve our understanding of what incentivises academics to practice Open Science.

## 3 Existing data sources

Our study compiles information on universities in terms of the PRT policies they adopt and their academic performance as measured by their research outputs. In this context, we survey the existing infrastructures which allow for extracting and processing the information we need to build our dataset.

### 3.1 Existing research output infrastructures

Large data corpora have been collected and become available from organisations, projects, commercial, and noncommercial services. These include:

- services that discover and deliver Open Access content,
- disciplinary repositories where Open Access content is submitted,
- harvesters that aggregate Open Access content available elsewhere,
- databases that use both Open Access and closed access content, and
- registries that provide essential information about the identification of the research outputs.

More specifically, Table 1 shows a non-exhaustive overview of the various resources available. In this work, we use MAG ^6^ and the CORE Discovery service ^7^ to compile dataset relevant to our needs. CORE Discovery hosts the largest collection of outputs, is the most recently released service as compared to alternative discovery tools and performs better both in content coverage and precision ^2^. In addition to that, some of the authors are affiliated with the service and are familiar with it. We use the 2018 release of the MAG database to extract the publication dataset for the research papers dataset. This situates the scope of our study to publications released until 2018, which is a fair choice considering the duration needed for the discovery of Open Access status of content from repositories. Both MAG and CORE Discovery are freely available for use and extracting data from both is straightforward, which makes them an ideal choice for our work.

**Table 1.** Research output infrastructure.

Resource name	Resource Type	Description	Free to use ^1^?	Collection Covered ^2^
CORE Discovery ^3^	Service	A tool that finds links to freely accessible research papers.	Yes	>24,936,921
OA Button ^4^	Service	A service that delivers links to an open access version of research articles (complemented by a request mechanism) by looking at thousands of resources.	Yes	This service uses a variety of data sources, which perform a “live” discovery and there is not a number available online.
Unpaywall ^5^	Service	An open database that harvests thousands of open access contents and delivers links to open access research articles.	Yes	26,009,865 free scholarly articles
Microsoft Academic Graph ^6^	Database	A multidisciplinary database consisting of scientific papers, demonstrates connections between papers and citations and offers rich metadata information, such as authors, institutions, journals, conferences and fields of study - free of cost service.	Yes	233 Million Paper Records
OpenAIRE Research Graph ^7^	Database	A multidisciplinary database of openly available scientific papers with additional information such as related datasets and software with funders, projects and communities	Yes	450 million metadata records
Scopus ^8^	Database	A multidisciplinary database of scientific papers in various disciplines such as social, life and health sciences	No	>34,000 journal titles
Web of Science ^9^	Database	Multidisciplinary databases with exhaustive citation data	No	>21,000 journal titles, 1.7 billion cited references
CrossRef	Registry	A registry of Digital Object Identifiers (DOIs)	Yes	Around 100 million metadata records from more than 4,500 publishers

^1^Some of these services may support products where payment is required. For this research, we used the free of cost products only. ^2^The numbers in the collection column are as of April 2020. ^3^CORE Discovery: https://core.ac.uk/services/discovery/ ^4^Open Access Button: https://openaccessbutton.org/ ^5^Unpaywall https://unpaywall.org/ ^6^Microsoft Academic Graph. https://www.microsoft.com/en-us/research/project/microsoft-academic-graph/ ^7^OpenAIRE Research Graph: https://www.openaire.eu/openaire-research-graph-open-for-comments ^8^Scopus: https://www.scopus.com/home.uri ^9^Web of Science: https://clarivate.com/webofsciencegroup/solutions/web-of-science/

### 3.2 Existing Universities’ Ranking Tools

The performance and quality of universities is often measured by using a mixture of factors. These focus on a variety of teaching, research performance and excellence components, collaborations with third parties, e.g., enterprise and industry, academic reputation, income, international student numbers, subject of field, and many more. Combining these elements yields comprehensive lists which, in turn, provide the universities’ ranking ^3^. Currently, there is a wide variety of national and international rankings, often supported by governments, newspapers, and websites. Table 2 provides some examples of the rankings used. All rankings weigh their indicators according to their own private algorithms to create the final ranking for each university.

**Table 2.** University ranking tools.

Resource name	Description	Topics
Times Higher Education World University Rankings ^9^ (THEWUR)	A global universities ranking list, examining institutions in five areas.	1. teaching 2. international outlook 3. industry income 4. research and 5. citations
Academic Ranking of World Universities ^10^ (ARWU)	A global universities ranking list, examining institutions in six areas.	1. # of alumni 2. total # of staff winning Nobel Prizes 3. # of highly cited researchers 4. # of papers published in Nature and Science 5. # of papers indexed in Science Citation Index-Expanded and Social Science Citation Index and 6. weighted scores of the above five indicators divided by the # of full-time equivalent academic staff
QS World University Rankings ^11^	A global universities ranking list, examining institutions in six areas.	1. academic peer review 2. faculty/student ratio 3. citations per faculty 4. employer reputation 5. international student ratio and 6. international staff ratio
UMultirank ^12^	A global universities ranking list, examining institutions in five areas	1. teaching and learning 2. research 3. knowledge transfer 4. international orientation and 5. regional engagement
UniversityRankings.ch ^13^	A global universities ranking list, examining institutions in two areas.	1. academic and 2. research performance
Round University Ranking ^14^	A global universities ranking list, examining institutions in four areas.	1. teaching 2. research 3. international diversity and 4. financial sustainability
The Carnegie Classification of Institutions in Higher Education ^15^	A university ranking tool focusing on U.S. Higher Education Institutions.	N/A
Macleans University Rankings ^16^	A university ranking tool focusing on Canada.	N/A

^9^THE World University Rankings: https://www.timeshighereducation.com/world-university-rankings/2020/world-ranking#!/page/0/length/25/sort_by/rank/sort_order/asc/cols/stats ^10^ARWU World University Rankings: http://www.shanghairanking.com/ ^11^QS World University Rankings: https://www.topuniversities.com/university-rankings/world-university-rankings/2020 ^12^UMultirank: https://www.umultirank.org/ ^13^Universityrankings.ch: https://www.universityrankings.ch/en ^14^Round University Ranking: https://roundranking.com/ranking/world-university-rankings.html#world-2019 ^15^Carnegie Classification: https://carnegieclassifications.iu.edu/ ^16^Rankings Archives: https://www.macleans.ca/education/unirankings/

Several studies have been conducted about university rankings. Aguillo *et al.* ^4^, compared various university ranking tools and discovered that despite the variety in their algorithms, they make use of similar attributes to compute the rankings. Pusser and Marginson ^5^ viewed the power of university rankings from a critical and theoretical point of view and found that these lists have an essential role in university power shaping. Saisana, d’Hombres and Salteli ^6^ discovered that, although at a country level the rating conclusions may not be as accurate, the results for larger scale areas are stronger.

The study from Alperin *et al.,* ^7^, relating to the dataset presented in this article, investigated the value of academia’s work by looking into USA and Canadian promotion review and tenure (PRT) policies, using the Carnegie Classification of Institutions in Higher Education and the Macleans University ranking to measure university scoring. The authors found that the current metrics relate more to ’classic and traditional’ evaluation components, such as publishing in subscription channels and citation metrics, and call for a shift change in the current assessment procedures. This work however uses a different university sample and only universities in the USA and Canada were considered.

## 4 Methods

### 4.1 Representative countries and universities selection

To conduct this research, there is the need to choose a single institutional ranking tool that would provide seamless access to the results of various countries globally. At the same time, this ranking should be normalised across countries, since the same indicators with the same weight should be applied. For these reasons, we use The World University Rankings (THEWUR) ^8^ as it includes *“global performance tables that judge research-intensive universities across all their core missions: teaching, research, knowledge transfer, and international outlook”* ^8^. In contrast to other similar tools, for example the Academic Ranking of World Libraries, THEWUR provides a direct indicator of how universities rank with regards to research and citations. Since the scope of this research is to look at PRT policies and connect them to research excellence and assessment, we choose THEWUR and two out of its five categories, as described below:

1. **Research**: Collecting data from the annual Academic Reputation Survey, this indicator is 30% of the total THEWUR. That excludes universities with less than 1,000 relevant publications between 2013 and 2017 and universities with 80% or more of their research outputs in a single subject area.
2. **Citations**: This indicator is 30% of the total THEWUR; the purpose of this category is to investigate the research impact of a publication, based on the number of times it is cited.

Next we describe how THEWUR is utilised for compiling our dataset, the reasoning behind the countries’ selection, and how we choose the universities in each country. We intend to select the same universities per countries as listed in THEWUR and investigate how they perform concerning the ’research’ and ’citation’ categories.

As the amount of national and institutional Open Access, open data ^9^ and in general Open Science policies vary per country ^10^, this work aims to investigate universities from a representative mix of countries from around the world. At the same time, this research is conducted by an international group of researchers, who speak and understand a variety of languages and can consequently collect policies in languages other than English. As a result, our dataset includes institutional policies written in three languages from seven countries.

### 4.2 PRT database

The number of institutions of the aforementioned countries varies significantly, i.e., there are countries with a large number of universities, e.g., Brazil, but also countries with much smaller numbers, e.g., Austria. For the PRT dataset, we initially selected a total of six universities per country (November 2019 to March 2020). At a later stage, or during the second round, (March 2021 to April 2021) the selection of the institutional policies was expanded. Due to the fact that the manual collection and analysis of policies is time-consuming We decided that the two rounds of policy collection would be sufficient to gain an understanding of our research question while making it feasible to conduct the research at the same time.

Our methodology for the collection of the six universities per country, as outlined below, ensures that the selection of universities is reproducible and that the outliers, i.e., universities performing extremely high or low in research and citations, are not included in the sample. Furthermore, the total number of universities is divided into three categories (high, medium, and low), and the median for each group is calculated. Within each category, we divide the total number of universities by three. In case the remainder is different from 0, we do the following:

- if the residual is 1, the first subcategory (high) will have 1 institution more than the other two (medium, low)
- if the residual is 2, then two subcategories (high and medium) will have 1 institution more

When within a subcategory there is an even number of universities, and their median is a decimal, we round down to the smaller integer value to select the median. When an institution would rank at the same position for both ’research’ and ’citations’, and in order to have a larger sample of policies, we choose the next available institution i.e., the one with the next highest rank in the category. If that institution is already included in the dataset or no policy is available for it, we pick the next lower ranking one.

The PRT policies are manually collected using a search engine. Table 3 shows the set of keywords identified and used for the policies identification in the three languages.

**Table 3.** Search key terms in English, German and Portuguese to retrieve related PRT policies.

English	German	Portuguese
policy	Satzung, Richtlinie, Verfahren	política de seleção, procedimento de seleção, procedimento, recrutamento
review	Qualifikationsprüfung, Review, Beurteilung, Leistungsevaluation, Regelung, Richtlinie, Strategie	revisão
academic, researcher, professor	wissenschaftliche Mitarbeiter, (Junior-) Professor	académico, universitário, investigador, professor
promotion	Beförderung, Promotion, Berufung	promoção

In the PRT dataset we include only institution-level policies due to difficulties in identifying specific departmental policies in some countries, for example the UK and USA. It is also challenging to assign policies on the universities’ websites to their specific departments. To ensure a consistent set of policies, we define the two exclusion rules: first, we do not collect advertisements of job descriptions even though these could include some insightful requirements applicable to the PRT policies. Second, PRT policies are not examined in conjunction with other policies that could relate to PRT, such as institutional policies about ethics or diversity and institutional open access policies.

The universities’ policies are then matched to a set of indicators. These include a selection of MoRRI, some indicators as mentioned in 7 and some that the authors believed to be prominent in the policies’ description during the research pilot. As a result, 18 different indicators are collected and examined (more information on the specific indicators can be found in Section 5.1). After the identification of both policies and indicators, we proceed with the cross-matching of these two components. To succeed in that, we go through each policy’s document and identify the sentence that discusses the respective indicator. We also extract the context, the before, and after sentences, and record them in the CSV file.

***4.2.1 Challenges in collecting PRT policies*.** Academic faculty policies are characterized by the diversity and the academic structures of respective countries. In the United Kingdom, for example, some universities have separate policies for associate research fellows, readers, professors and full professors. In contrast, in the United States, oftentimes separate policies are created for tenured and non-tenured staff and while the same applies for Portugal and Brazil. In Austria, policies either refer to ’habilitation’, i.e., a qualification for teaching that is essential for promotion to professor, or to qualification agreements (tenure track) for associate professors, whereas calls do not include promotions to full professors. The German policies refer to the English term of ’tenure track’ and specify the evaluation process, including details on the committee and the evaluation criteria. The authors are local experts and used specific terminologies in the promotion policies for each country while searching for the policies on the web.

In addition, there were policies which could be accessed freely via the internet, but a limited amount could be found behind a username and password. Those not openly accessible policies that required an institutional log-in, i.e., four policies from Austrian universities, two Brazilian, and a British, are obtained after contacting universities *via* email requesting for a copy.

### 4.3 Research data papers dataset

***4.3.1 Data source*.** For this dataset, we make use of MAG and CORE Discovery. MAG is organized into database tables that provide a variety of information on scholarly publications such as citations, author names, institution names (universities as well as other publishing bodies), and publication years ^11^. We use CORE Discovery to determine the Open Access status of the scholarly publications retrieved from MAG. Given a DOI for a paper as input, CORE Discovery returns a flag which indicates whether the paper is known to be Open Access or of unknown status ^12^. Since we use the 2018 release of the MAG database, our data contains records of publications as recent as 2018 and this consists of all publication types as available on MAG, i.e., conference or journal articles, review papers, book chapters etc. Further, this dataset records data for all universities identified in THEWUR and common to MAG for the countries in our sample and not just the universities used for the PRT dataset. This is designed to facilitate the dataset reuse for analysis of universities with and without promotion policies in comparison to their publication output. With regards to the career profile of academics, we obtain the information on authors’ profile by consuming the information directly available on MAG; e.g., authors rank, total papers count, total citation counts, as well as by further processing of related information, e.g., determining the seniority of authors based on the number of years since first publication until their last publication. Other potential sources for obtaining career profiles, e.g., LinkedIn, Google Scholar, ORCID, have not been explored in the course of this work as such resources are not freely and easily available for use.

***4.3.2 Dataset creation methods*.** To create the research data papers dataset we identified six research questions that could assist with the understanding of the publishing research behaviour at an institutional level and provide insights, which are not available only by analysing the PRT data. These questions are:

1. What percentage of papers coming from an institution is Open Access ?
2. How are papers published by the universities distributed across the three scientific disciplines of our choice – Agriculture, Climatology and Medicine?
3. What is the gender distribution in the authorship of papers published by the universities?
4. What is the distribution of seniority, i.e., number of years since first publication until last publication, of staff in the universities?
5. What is the distribution of incoming citations for Open Access versus other papers published by the institution ? In other words, if institution **A** publishes **X** number of Open Access papers and **Y** number of papers for which their open access status is unknown; what is the count of citations received for **X** vs **Y**.
6. What is the distribution of references made for Open Access articles versus other papers in articles published by the universities, i.e., if institution **A** publishes **X** papers which reference **M** papers; how many of those **M** papers are Open Access vs unknown.

Questions 1,5,and 6 relate to the use of Open Access in the targeted institutions, both as a publishing practice and as a citation behaviour. Questions 3 and 4 describe the researcher population of the institution and question 2 helps understanding how the institution fits against the predefined scientific disciplines.

Table 4 outlines our usage of CORE Discovery, MAG and other external sources to create this dataset.

**Table 4.** Research data papers dataset: Data sources used and output data.

Question no.	Microsoft Academic Graph (MAG) Tables Used	CORE Discovery Used (Y/N)?	External Data Used and its Purpose	Output Data Schema
1	Papers, Affiliations, PaperAuthorAffiliations	Yes	Natural Earth Dataset to map geographic coordinates of institutions.	PaperID, Univ_name, Country_name, OA_flag
2	Papers, Affiliations, PaperAuthorAffiliations, PaperFieldsofStudy, FieldsofStudy	No	Same as for Question No. 1	PaperID, Univ_name, Country_name, FieldofStudy
3	Papers, Affiliations, PaperAuthorAffiliations, Authors	No	Same as for Question No. 1 + Gender API for gender detection of authors.	PaperID, Univ_name, Country_name, Author_Name, Gender
4	Papers, Affiliations, PaperAuthorAffiliations	No	Same as for Question No. 1	PaperID, Univ_name, Country_name, AuthorID, Year
5	Papers, Affiliations, PaperAuthorAffiliations	Yes	Same as for Question No. 1	PaperID, Univ_name, Country_name, Citation_Count, OA_flag
6	Papers, Affiliations, PaperAuthorAffiliations, PaperReferences	Yes	Same as for Question No. 1	PaperID, Univ_name, Country_name, Count_OA_References, Count_Unknown_References

***4.3.3 Challenges in creating the dataset*.** There are several challenges involved in creating this dataset. To begin with, processing the MAG database calls for techniques in big data processing and needs appropriate hardware support in a cluster computing environment. Analysing such data in conjunction with CORE Discovery and other external resources is a multi-step task which requires efficient resource planning and software optimization. There are issues with the universities in THEWUR not matching to the institution names in MAG, and we perform text normalisation (lowercase, punctuation removal, and ASCIIfication) on the universities’ names to look for a match. We also take proper care to discard duplicates seen within the collection for the same institution . That applies, for example, ensuring that the same paper (PaperID) is not recorded twice in our dataset with the OA_flag set to true because there are two entries in MAG for that paper; each one being associated to one of the two distinct co-authors from the same institution for that paper. On the contrary, a paper could have multiple authors affiliated with different universities. In such cases, we included a single instance of the paper for each of the universities concerned.

## 5 Results

### 5.1 PRT Dataset Details

As discussed earlier, the PRT dataset consists of data from seven countries, across 107 universities per country. This information is presented in a CSV file, which contains the following fields:

1. **institution_name:**: name of the institution.
2. **institution_policy_id**: Identifier for the policy. Format: XX_n, where "XX" is a country code such as "AT", "UK" or "USA", and "n" is a random number referring to a specific institution. Finally, some institutions had more than one policy, in which case we added lowercase letters (a, b, c).
3. **indicator_group**: Indicator/criteria group. These are categories for the column "indicator_code".
4. **indicator_code**: Indicator code. Code to identify a particular indicator.
5. **indicator_question**: Description of the indicator.
6. **indicator_value**: The result of coding the documents for presence (1) or absence (0) of an indicator/criterion.
7. **qual_answer**: The text fragment which was relevant to the coding, i.e., text pasted from the policy itself. This was omitted for policies from Austria due anonymity constraints.
8. **english_version**: translation of the non-English content, based on Google translate.
9. **qual_answer_2**: This contains additional text, e.g. where multiple options were present in the document (as for the assessed positions).
10. **comment**: Contains comments made during data collection, relating to the decisions regarding the coding of the indicators/criteria.
11. **data_collection_round**: Information on when the policy was collected, i.e. during the first round (November 2019 to March 2020) or during the second round (March 2021 to April 2021).
12. **affiliationid**: enables matching the institution with the corresponding publishing production in the Research Papers dataset.

With regards to the policy indicators’, where an indicator is marked with zero, it would not apply and when with one it would be applicable. Table 5 reports the number of policies analysed per country and the percentage of institutions where the respective indicators were observed.

**Table 5.** Indicator quantities per country.

	Austria	Brazil	Germany	India	Portugal	UK	USA
Policies Count	13	15	13	12	7	48	36
Gender Equality	67%	0%	42%	0%	0%	0%	0%
Gender Reviewers	50%	0%	58%	0%	0%	0%	0%
Gender Balance Reviewers	33%	0%	33%	0%	0%	0%	0%
Citizen Science	0%	8%	8%	0%	0%	0%	6%
Public Engagement	17%	42%	25%	8%	100%	62%	17%
Policy Makers	17%	33%	8%	0%	0%	54%	14%
Industry	33%	33%	33%	25%	83%	62%	20%
Open Access	0%	0%	0%	0%	0%	0%	0%
Data	0%	0%	0%	0%	0%	0%	0%
Software	0%	75%	8%	0%	0%	0%	11%
Journal Metrics	50%	42%	25%	67%	17%	12%	14%
Citations	17%	0%	33%	8%	0%	17%	26%
Number of publications	67%	25%	25%	8%	33%	4%	17%
Publication quality	33%	0%	58%	33%	17%	12%	14%
Peer review	17%	75%	15%	0%	50%	58%	40%
Pastoral work	50%	100%	33%	58%	83%	100%	63%
Patents	33%	75%	67%	67%	67%	4%	34%

### 5.2 Research paper dataset details

Table 6 shows the count of total universities in THEWUR and the corresponding matches we found in MAG. This accounts for a total of 379 universities included in our dataset. The lowest coverage is seen for Germany (70.83%) while others have fairly good coverage, with the United Kingdom having the highest (91.0%). The difference in coverage can be linked to the difference in the data sources THEWUR uses (possibly Scopus and others) compared to our choice of MAG. In total, the dataset contains information on 126,795 distinct papers published from the universities in Austria, 682,819 from Brazil, 664,165 from Germany, 272,784 from India, 139,983 from Portugal, 1,490,843 from the United Kingdom, and 4,844,193 from the United States.

**Table 6.** Count of Universities found in the World University Rankings (THEWUR) and Microsoft Academic Graph (MAG) for each country analysed.

	Austria	Brazil	Germany	India	Portugal	UK	USA
THEWUR count	11	46	48	56	13	100	172
MAG Count	9	34	34	46	11	91	154
% coverage	81.81	73.91	70.83	82.14	84.61	91.0	89.53

## 6 Discussion and future work

Promotion policies are an important lever when trying to change researcher behaviour. They make the current norms of the scientific system explicit and determine who will be able to continue their career within academia by rewarding certain practices. Current initiatives like the Hong Kong Principles for Assessing Researchers ^9^ aim at increasing the trustworthiness of research through recognising practices such as responsible research, transparent reporting and Open Science *via* the promotion of policies. Our dataset will enable other researchers to investigate how certain open science aspects mentioned in promotion policies are associated with researchers’ productivity. Our dataset opens new avenues for future research; we present example research questions for which our dataset can be used below:

1. If an institution mentions “number of publications” as a requirement in its promotion policy,
  (a) How high does it rank in terms of the overall share of Open Access publication it produces?
  (b) How often do papers published by that institution reference papers which are Open Access?
  (c) Do authors affiliated with that institution get a higher number of citations, on average?

2. For an institution that has a gender equality requirement in its promotion policy, what is the gender distribution in the authorship of papers published by that institution ?

3. Does the number of years that authors have to remain at a specific grade level before being eligible to apply for a higher grade level correlate with the number of years they usually stay at an institution?

4. How do the representative universities rank in terms of research output performance compared to other universities for which the promotion policy could not be found?

Question 1.a, for example, could be answered by analysing the ’number of publications’ published by universities that mention the ’number of publications’ in the PRT policies. Identifying further questions and interpreting their results lies within the scope of future work that can be carried out with our datasets.

Research achievement should be assessed in two ways: 1. in terms of its impact on the research community and society; and 2. with regards to the originality of ideas and research integrity. Unfortunately, other external factors, e.g., personal characteristics or commonly held opinions of a person’s character, could erroneously also sometimes negatively impact this practice. The new concepts, Open Science and RRI, aim to promote the conduction of research, through public participation and make it more inclusive, participatory, understandable, accessible and re-usable. These concepts can bring a change and have an effect only when countries, universities and policy-makers are in position to absorb and integrate them in the current research assessment policies. Our study is working towards this direction, i.e., showcasing the low uptake of Open Science practices in the PRT policies, and consequently in the universities around the world, and urge for their higher adoption.

Our dataset could have been much richer in terms of the PRT data if the individual policies were themselves originally available in a structured machine-readable format, e.g., in XML and documented using a common standard. In that case, our CSV dataset fields could carry specific information about core aspects of the policies. For example, current policies often do not specify when the policy was created, applied or amended. Should such information be available, then the higher level of detail would give us more complete data and enable the drawing of more informed conclusions, producing more thorough and compelling results. This could also enable making connections and identifying the emergence of new fields in the policies relating to open science and RRI. When these components are added in the policies, these could then be easily processed by machines, enabling an automatic comparison among them. In that case, conclusions could be easily drawn as to whether a PRT policy from institution A promotes pen Science and RRI components as compared to a policy from institution B.

A limitation with regards to our datasets also arises from the fact that the Open Access availability of certain research outputs are realised only after an embargo period. In this research, we took into consideration the status of the research outputs as of April 2020, without an effort to determine their Open Access availability at the time of their first publication. Because of this, estimates for the propensity of Open Access are likely higher than if they were based on data on Open Access status at the publication date. In addition, the policies’ publication time in the PRT dataset could sometimes be unknown or outdated. As a result, further work is needed for assessing causality between indicators. Finally, PRT policies are not the sole factor for promotion; the diversity of processes relating to career progression per institution, per country and their interconnection to universities’ strategy and culture can also affect the process.

## 7 Conclusion

In this article, we presented the dataset which consists of two parts: 1. a collection of promotion, review, and tenure policies from universities in seven countries; and 2. a research papers dataset, consisting of publication records for universities listed in THEWUR for the same countries, which can be associated using a shared identifier. The significance of this dataset is that it allows for a quantitative study of questions related to academic productivity, Open Science practices and incentives. During the curation of this dataset from multiple sources, it became evident that there is a need for machine accessibility of institutional policies, so that the policies’ descriptions can be classified more easily and taken into account when investigating pen Science and RRI uptake based on large scholarly corpora. The dataset has already been used in the ON-MERRIT project and will also be utilised on the project’s several forthcoming publications.

## Data availability statement

*Underlying data:-* The Open University: Career promotions, research publications, Open Access dataset.

https://doi.org/10.21954/ou.rd.19228785.v1.

The project contains the following underlying data:

- Dataset.zip (The dataset is a combination of two different data sources, one part is a dataset created on analysing promotion policies across the target countries, while the second part is a set of data points available to understand the publishing behaviour. ).

Data are available under the terms of the Creative Commons Attribution 4.0 International license (CC-BY 4.0).

## Footnotes

^1^Open Science is the movement to make scientific research, data and dissemination accessible to all levels of an inquiring society. https://www.fosteropenscience.eu/taxonomy/term/7

^2^In June 2021 MAG was discontinued https://www.natureindex.com/news-blog/microsoft-academic-graph-discontinued-whats-next and currently alternative initiatives are being examined to fill the gap

^3^Open-access (OA) literature is digital, online, free of charge, and free of most copyright and licensing restrictions. What makes it possible is the internet and the consent of the author or copyright-holder. https://legacy.earlham.edu/ peters/fos/brief.htm

^4^ https://on-merrit.eu

^5^Responsible Research and Innovation is: Involving society in science and innovation ‘very upstream’ in the processes of R&I to align its outcomes with the values of society. A wide umbrella connecting different aspects of the relationship between R&I and society: public engagement, Open Access, gender equality, science education, ethics, and governance. (Definition from https://www.rri-tools.eu/about-rri)

^6^The MAG service was retired in November 2021

^7^ https://core.ac.uk/services/discovery

^8^THE World University Rankings: https://www.timeshighereducation.com/world-university-rankings/2020/world-ranking#!/page/0/length/25/sort_by/rank/sort_order/asc/cols/stats

^9^Open Data is data that can be freely used, re-used, and redistributed by anyone - subject only, at most, to the requirement to attribute and sharealike. https://opendatahandbook.org/guide/en/what-is-open-data/

^10^ROARMap http://roarmap.eprints.org/

^11^The complete list of all MAG database tables as well as their is available at https://tinyurl.com/v4r5tfv

^12^CORE Discovery may not have a 100% Open Access discovery success, for example in case of a missing DOI; hence we can only say that such papers have an ’unknown’ status.
