# Supplementary material for: How do career promotion policies affect research publications and open access?
Source: Open Res Eur. 2022 Aug 22;2:99. [Version 1] doi: 10.12688/openreseurope.14921.1 (PMC11426022; doi:10.12688/openreseurope.14921.1)
Supplement: Peer review history

# Reviewer report: Reviewer response for version 1

**DOI:** 10.21956/openreseurope.16125.r32638

**Published:** 2024-09-24

**Authors:** Stuart Buck

**Affiliations:** 1 Good Science Project, Houston, TX, USA

This article is primarily to present a new dataset constituting the "promotion, review, and tenure policies (PRT) from institutions originating from seven countries and combined them with bibliographical data from the outputs generated by each institution." The dataset covers 379 institutions across those seven countries. The dataset obviously took a huge amount of manual collection and curation, and represents a leap forward in the availability of such data across many institutions and countries. I therefore expect it to be a useful addition to future studies on open science practices and policies in many fields.

If applicable, is the statistical analysis and its interpretation appropriate?

Not applicable

Is the study design appropriate and is the work technically sound?

Yes

Is the work clearly and accurately presented and does it engage with the current literature?

Yes

Are the conclusions drawn adequately supported by the results?

Yes

Are sufficient details of methods and analysis provided to allow replication by others?

Yes

Are all the source data and materials underlying the results available?

Yes

Reviewer Expertise:

Open science

I confirm that I have read this submission and believe that I have an appropriate level of expertise to confirm that it is of an acceptable scientific standard.

# Reviewer report: Reviewer response for version 1

**DOI:** 10.21956/openreseurope.16125.r33791

**Published:** 2023-07-31

**Authors:** Pablo Sastrón-Toledo

**Affiliations:** 1 Department of Library and Information Sciences, Carlos III University of Madrid, Madrid, Spain

This article provides a detailed explanation of two novel datasets whose information can be greatly valuable to analyze research performance regarding RRI criteria. Congratulations to the authors. This topic is of particular relevance in the European context and I am sure this data will arouse the interest of researchers in the scientific community. However, I believe that the work may improve in some aspects. I hope that the authors find my comments helpful.

**General comments:**

- I think the paper would benefit from having an explanation of MoRRI indicators (the first time they appear is in section 2). It might also be interesting to include a brief explanation of the ON-MERRIT project so the reader understands better the context surrounding the data.
- I believe section 2 is an introduction to section 3. Section 2 provides an overview of the data and section 3 describes which resources are better to build the data. Maybe merging these sections is useful to the reader? Some section 2 statements are built on information provided in section 3 (i.e. such as MAG and CORE being used as the core databases for the research papers dataset). If you don´t feel comfortable merging it I would then suggest to include some context information in section 2 to clarify.
- The dataset is composed of two subsets: one dedicated to PRT and another dedicated to research papers. In section 3, there is a description of these subsets. The PRT dataset is explained later in section 4.2. I would suggest including this description (the one in 4.2) in section 3. Also, I would describe more explicitly the aim of using university rankings to select the unified data from institutions; perhaps at the beginning of 3.2.
- Table 1 Resource Type taxonomy seems a bit confusing to me, categories overlap. I think all the resources are databases and services (Unpaywall, as you refer on the description; CrossRef, CORE Discovery and OA Button). The main differences I see is that some are open (Unpaywall, MAG, Crossref, OpenAIRE, CORE Discovery, OA Buton), some other private (WoS and Scopus). I would appreciate a more detailed explanation of the differences between the resources taxonomy.

**About style and consistency:**

- Some specific terms related to open science such as open data, open access, RRI are briefly explained in footnotes. I would rather set an explanatory introduction on how this work is related to open science and its different dimensions. This would avoid footnotes and I think that the work would benefit from a short introduction on this topic.
- Some key terms are treated unevenly in the paper i.e. open science is written in upper case/lowercase indistinctly.
- In footnote 10 related to ROARMap, I would suggest explaining briefly how ROARMap is a resource that may help identify the degree of development of open science policies across countries.
- Footnote 11 ['The complete list of all MAG database tables is available...'] link does not work.
- I am not sure that the acronym PRT (for promotion, review, and tenure policy) has been used widely so I would suggest including at the beginning that you are naming it like that for readability reasons i.e. […] for promotion, review, and tenure policy (PRT hereafter).
- Sometimes is tricky to distinguish between references and footnotes. I would suggest using separate marks for each of them.

If applicable, is the statistical analysis and its interpretation appropriate?

Not applicable

Is the study design appropriate and is the work technically sound?

Partly

Is the work clearly and accurately presented and does it engage with the current literature?

Partly

Are the conclusions drawn adequately supported by the results?

Yes

Are sufficient details of methods and analysis provided to allow replication by others?

Partly

Are all the source data and materials underlying the results available?

Yes

Reviewer Expertise:

Open science, open access

I confirm that I have read this submission and believe that I have an appropriate level of expertise to confirm that it is of an acceptable scientific standard, however I have significant reservations, as outlined above.

# Reviewer report: Reviewer response for version 1

**DOI:** 10.21956/openreseurope.16125.r33793

**Published:** 2023-07-31

**Authors:** Rob Johnson, Eleanor Cox

**Affiliations:** 1 Research Consulting Ltd, Nottingham, UK

The article is a presentation of a methodology and dataset. We found there were many areas where the research was well formulated and the underlying dataset was both easily accessible and comprehensible. The international nature of the study and the size of the research data papers dataset are notable strengths. However, there were areas in which some of the underlying assumptions are stated without adequate recognition of their complex and contested nature. For example:

- Concepts such as research behaviour and performance (section 1) and the productivity of academics (section 2) are introduced uncritically, together with the presumption that these can be captured and measured quantitatively. The article would benefit from some acknowledgement of the potential limitations of quantitative measures and their variable applicability to different academic disciplines.
- There appears to be an a priori assumption in the paper that Open Science and Responsible Research and Innovation (RRI) factors can influence academic progression and tenure. While this is not unreasonable, it would be helpful for the authors to explain why this might be the case, and reference existing work that leads them to this conclusion and provides a clear rationale for this study.
- The authors state in section 6 that research achievement should be assessed in two ways but do not state why they believe this, nor do they evidence this with any relevant literature on the topic.
- The article examines university rankings in representative countries and universities. However, the references to rankings are noticeably dated and article does not engage with relevant arguments or literature concerning whether ranking universities is a helpful exercise in determining their worth as institutions. An engagement with regarding the reservations regarding ranking and an explanation of the reasoning for using ranking systems would strengthen this area. See, for example, Barnett, Adrian and Gadd (2022) ^1^ and Gadd (2020). ^2^

Section 2 on the dataset significance requires some refining to explain to the reader the importance of the study and explain what benefits might be derived from establishing a relationship between Open Science and Responsible Research and Innovation (RRI) factors and the productivity of academics. Without this, and in the absence of any empirical findings, it is hard to assess the dataset’s significance. Further, a fuller acknowledgment that this work is just a small part of a larger body of evidence surrounding promotion in higher education and that there are many other factors alongside PRT policies, institutional strategies and culture which affect tenure would be helpful. We also believe that moving the research questions to this section would strengthen understanding in what the dataset is for.

 We found that the title of the article is misleading. Currently, the title implies that the question regarding promotional policies affecting research publication and open access is going to be answered in the text, which it is not. Rather, the title needs reframing to highlight that the article focuses on the dataset and the process of its creation.

 We found that Section 4.2 was neither comprehensible nor easily reproducible. The methodology is unclear regarding the process of selecting universities. The authors make reference to a second round of institutional policy selection where the choice of universities was expanded but do not state how many more universities was chosen, or what made the second round different from the first. The authors do not define what the categories high, medium and low mean, without which the following explanation about residuals is meaningless.

 We also believe that the authors should address some more minor comments:

- Throughout the paper, there is reference to the need for the machine readability of PRT policies. The paper gives no consideration to how such a change might be implemented (for example the need to develop an internationally-accepted schema for this purpose) and the barriers and incentives associated with such a change.
- More context is needed regarding what PRT policies look like and what information they contain.
- An explanation as to what MoRRI factors are or what the acronym stands for is needed.
- There is some confusion regarding the number of universities and policies actually looked at. A clear statement of the total number of policies and universities is needed.
- The authors choose the disciplines of agriculture, climatology and medicine to focus on but do not provide the rationale behind this. This rationale needs to be clearly stated.
- Some minor grammatical and spelling mistakes throughout.

**References:**

^1^ Barnett, Adrian & Gadd. 2022. University league tables have no legs to stand on. Significance, 19 (4), 4-7. https://rss.onlinelibrary.wiley.com/doi/epdf/10.1111/1740-9713.01663

^2 ^Gadd. 2020. University rankings need a rethink. *Nature. *587, 523. https://doi.org/10.1038/d41586-020-03312-2

If applicable, is the statistical analysis and its interpretation appropriate?

Not applicable

Is the study design appropriate and is the work technically sound?

Partly

Is the work clearly and accurately presented and does it engage with the current literature?

Partly

Are the conclusions drawn adequately supported by the results?

Yes

Are sufficient details of methods and analysis provided to allow replication by others?

Partly

Are all the source data and materials underlying the results available?

Yes

Reviewer Expertise:

research management, open access, bibliometrics

We confirm that we have read this submission and believe that we have an appropriate level of expertise to confirm that it is of an acceptable scientific standard, however we have significant reservations, as outlined above.

## References

[1] : University League Tables have no Legs to Stand on. *Significance* .2022;19(4) : 10.1111/1740-9713.01663 4-7 10.1111/1740-9713.01663 35601695

[2] : University rankings need a rethink. *Nature* .2020;587(7835) : 10.1038/d41586-020-03312-2 523 10.1038/d41586-020-03312-2 33235367

# Reviewer report: Reviewer response for version 1

**DOI:** 10.21956/openreseurope.16125.r29954

**Published:** 2022-10-12

**Authors:** Nicolas Robinson-Garcia

**Affiliations:** 1 EC3 Research Group, Information and Communication Studies Department, University of Granada, Granada, Spain

This data paper describes two interesting datasets which have been made publicly available. I congratulate the authors for their effort as this type of information is very valuable and difficult to obtain. However I believe there is still room for improvement. Following I include my suggestions and comments:

- There is confusion between endnotes and references, the same marks are being used.
- I think a bit of context is needed to the reader. I suggest adding a section between 1 and 2 in which some background information is given to the reader regarding the concepts of Open Science and RRI
- Also, some of the acronyms used are provided without context. What are MoRRI indicators?
- Probably the section 2 should be provided later on in the paper, as it discusses many things which have not been explained to the reader. What is MAG, what is the CORE Discovery service (these are described, yes, but later in the text), what do you mean by PRT policies?
- In section 3.1 you refer to Open Access content, do you refer all the time to scientific publications?
- Some more details on description, coverage and metadata on MAG and CORE Discovery are needed.
- In section 3.2 you talk about excellence components, what do you mean by that? Which rankings are supported by governments?
- The review on university rankings is extremely superficial and outdated, the justification for using the THEWUR is quite vague. This ranking has been actually seriously criticized, especially the survey data, which is heavily biased favouring UK institutions. No mention to more rigorous rankings such as the Leiden Ranking are made nor to what exactly rankings are portraying. See for instance Robinson-Garcia *et al.* (2019) ^1^; Selten *et al.* (2020) ^2^; and Moed (2017) ^3^ In the context of RRI I have serious doubts that including such information is useful.
- Page 7, last paragraph before section 4.2.1. Further information on the indicators selected and the rationale for selecting such indicators is needed.

If applicable, is the statistical analysis and its interpretation appropriate?

Not applicable

Is the study design appropriate and is the work technically sound?

Partly

Is the work clearly and accurately presented and does it engage with the current literature?

Partly

Are the conclusions drawn adequately supported by the results?

Yes

Are sufficient details of methods and analysis provided to allow replication by others?

Partly

Are all the source data and materials underlying the results available?

Yes

Reviewer Expertise:

university rankings, open access, scientometrics

I confirm that I have read this submission and believe that I have an appropriate level of expertise to confirm that it is of an acceptable scientific standard, however I have significant reservations, as outlined above.

## References

[1] : Mining university rankings: Publication output and citation impact as their basis. *Research Evaluation* .2019;28(3) : 10.1093/reseval/rvz014 232-240 10.1093/reseval/rvz014

[2] : A longitudinal analysis of university rankings. *Quantitative Science Studies* .2020;1(3) : 10.1162/qss_a_00052 1109-1135 10.1162/qss_a_00052

[3] : A Comparative Study of Five World University Rankings. 10.1007/978-3-319-60522-7_18 261-285 10.1007/978-3-319-60522-7_18
